# Effect of Aggregation and Molecular Size on the Ice Nucleation Efficiency of Proteins

**DOI:** 10.1021/acs.est.3c06835

**Published:** 2024-02-26

**Authors:** Alyssa N. Alsante, Daniel C. O. Thornton, Sarah D. Brooks

**Affiliations:** † Department of Oceanography, Texas A&M University, College Station, Texas 77843, United States; § Department of Atmospheric Sciences, Texas A&M University, College Station, Texas 77843, United States

**Keywords:** aerosol, clouds, ice-nucleating particle, immersion freezing, protein aggregation

## Abstract

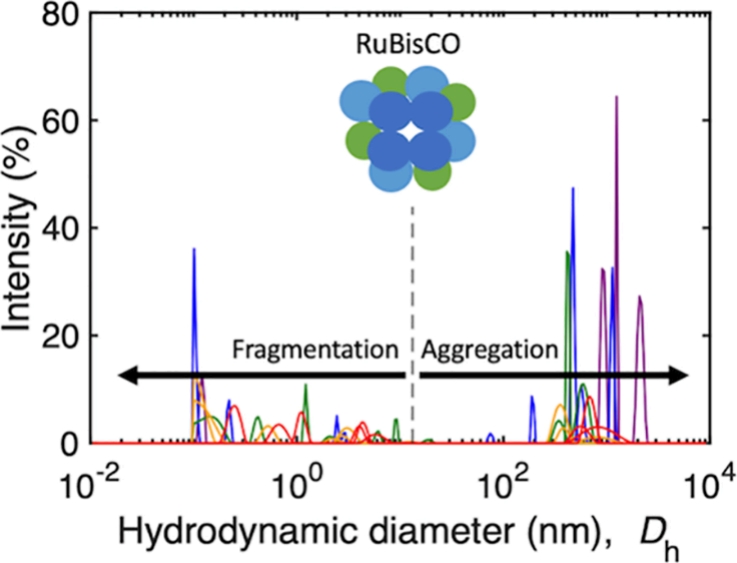

Aerosol acts as ice-nucleating particles (INPs) by catalyzing the formation of ice crystals in clouds at temperatures above the homogeneous nucleation threshold (−38 °C). In this study, we show that the immersion mode ice nucleation efficiency of the environmentally relevant protein, ribulose-1,5-bisphosphate carboxylase/oxygenase (RuBisCO), occurs at temperatures between −6.8 and −31.6 °C. Further, we suggest that this range is controlled by the RuBisCO concentration and protein aggregation. The warmest median nucleation temperature (−7.9 ± 0.8 °C) was associated with the highest concentration of RuBisCO (2 × 10^–1^ mg mL^–1^) and large aggregates with a hydrodynamic diameter of ∼10^3^ nm. We investigated four additional chemically and structurally diverse proteins, plus the tripeptide glutathione, and found that each of them was a less effective INP than RuBisCO. Ice nucleation efficiency of the proteins was independent of the size (molecular weight) for the five proteins investigated in this study. In contrast to previous work, increasing the concentration and degree of aggregation did not universally increase ice nucleation efficiency. RuBisCO was the exception to this generalization, although the underlying molecular mechanism determining why aggregated RuBisCO is such an effective INP remains elusive.

## Abstract 2

Atmospheric ice nucleation remains a major uncertainty in understanding cloud processes and climate. This study reports the effects of molecular size and aggregation on the immersion mode ice nucleation efficiency of proteins.

## Introduction

Ice crystals are important in clouds at all latitudes, affecting Earth’s radiative budget and hydrological cycle.^1,2^ Aerosol may act as ice-nucleating particles (INPs), catalyzing heterogeneous ice crystal formation at temperatures warmer than homogeneous freezing of pure water droplets (−38 °C).^3,4^ INPs represent 1 in 10^5^ (or fewer) aerosol particles in the troposphere but exert significant influence on cloud microphysical processes.^5^ The sources and composition of INPs are not well-understood, and their effects on cloud properties remain the largest source of uncertainty in climate modeling.^1,6^

The most well-known class of INPs are mineral dust, but mineral dust cannot account for INPs observed to catalyze freezing at > –15 °C. Warmer ice nucleation activity is mostly attributed to biological INPs.^3,7,8^ Biological sources of INPs include pollen,^9−13^ phytoplankton,^14−17^ bacteria,^18−21^ archaea,^22^ fungi,^23−25^ and viruses.^26,27^ Most previous work has investigated whole organisms and extracellular organic matter as INPs,^14,28,29^ with individual compounds gaining more recent attention. Individual biomolecules effective as INPs include cell wall components, such as lignin^30^ and cellulose,^31,32^ amino acids,^33,34^ polysaccharides,^34^ lipids,^34^ nucleic acid,^33^ and proteins.^20,26,33−36^

Microbially produced INPs from plant-associated bacteria, including *Pseudomonas syringae*, are some of the most catalytically efficient INPs known, and they are ubiquitous in the atmosphere.^37,38^ InaZ, the INP from *P. syringae*, has an onset freezing temperature of −1.8 °C and is completely ice-active by −12 °C.^20,36^ A 10-fold dilution series (from 10^0^ to 10^–7^ mg mL^–1^) of *P. syringae* showed it has high onset freezing temperatures of > –10 °C until a low concentration (≤10^–6^ mg mL^–1^), where it does not activate until < –20 °C. The low freezing temperature at a low concentration is potentially due to a lower degree or absence of molecular aggregation.^38^

Other large proteins are known to be effective INPs. Apoferritin and ferritin (>400 kDa) are ice-active at temperatures of > –10 °C, with large aggregates contributing to the highest observed ice nucleation efficiency.^26^ Ribulose 1,5-biphosphate carboxylase/oxygenase (RuBisCO, ∼550 kDa^39^) induces freezing at temperatures as high as −6.8 °C, which is warmer than most other currently known INPs.^33^ In addition, RuBisCO is one of the most abundant proteins in both terrestrial and marine environments because it is an essential photosynthetic enzyme in plants and phytoplankton.^40^ It has been isolated from ambient terrestrial aerosol (up to 9.1 × 10^–6^ ice nucleation active sites L^–1^ of air at −9 °C), with potential global implications for Earth’s radiative budget and climate.^33^

Collectively, these findings suggest that size may play an important role in determining the ice nucleation efficiency of proteins, with large protein molecules and protein aggregates being highly effective INPs. To test this hypothesis, we investigated the role of the molecular size as well as the aggregation of proteins in immersion freezing across a wide range of concentrations. Immersion freezing occurs when an INP is immersed in a liquid droplet and is the most common mechanism of heterogeneous ice nucleation observed in the atmosphere under tropospheric conditions.^3,5^ To measure aggregation, we used dynamic light scattering (DLS) to complement the ice nucleation measurements. The proteins selected ranged in molecular weight from 5.8 to 550 kDa and were measured over concentrations spanning 5 orders of magnitude (from 2 × 10^–1^ to 2 × 10^–5^ mg mL^–1^).

## Methods

### Sample Choice and Preparation for Ice Nucleation and DLS Experiments

Individual proteins (RuBisCO, pyruvate kinase, alkaline phosphatase, lipase, and insulin) and a peptide (glutathione) were chosen based on differences in molecular weight and structural features. A known mass of each protein was diluted using ultra-high-performance liquid chromatography (UHPLC) water using a 10-fold dilution series from 2 × 10^–1^ to 2 × 10^–5^ mg mL^–1^. The diluted samples were stored in 1.5 mL microcentrifuge tubes for up to 1 week at 4 °C prior to ice nucleation analysis and analyzed immediately after dilution for DLS. The samples were shaken prior to measurement to ensure homogeneity. Each sample was obtained from a commercially available compound (Sigma-Aldrich). RuBisCO was isolated from spinach (*Spinacia oleracea*) and is representative of the most abundant type (form I) on Earth.^41^ It was used in a previous ice nucleation study.^33^ Pyruvate kinase originated from rabbit (*Oryctolagus cuniculus*) muscle, with alkaline phosphatase from bovine (*Bos taurus*) intestine, lipase from fungi (*Aspergillus niger*), and insulin from the bovine pancreas.

**Figure 1:**
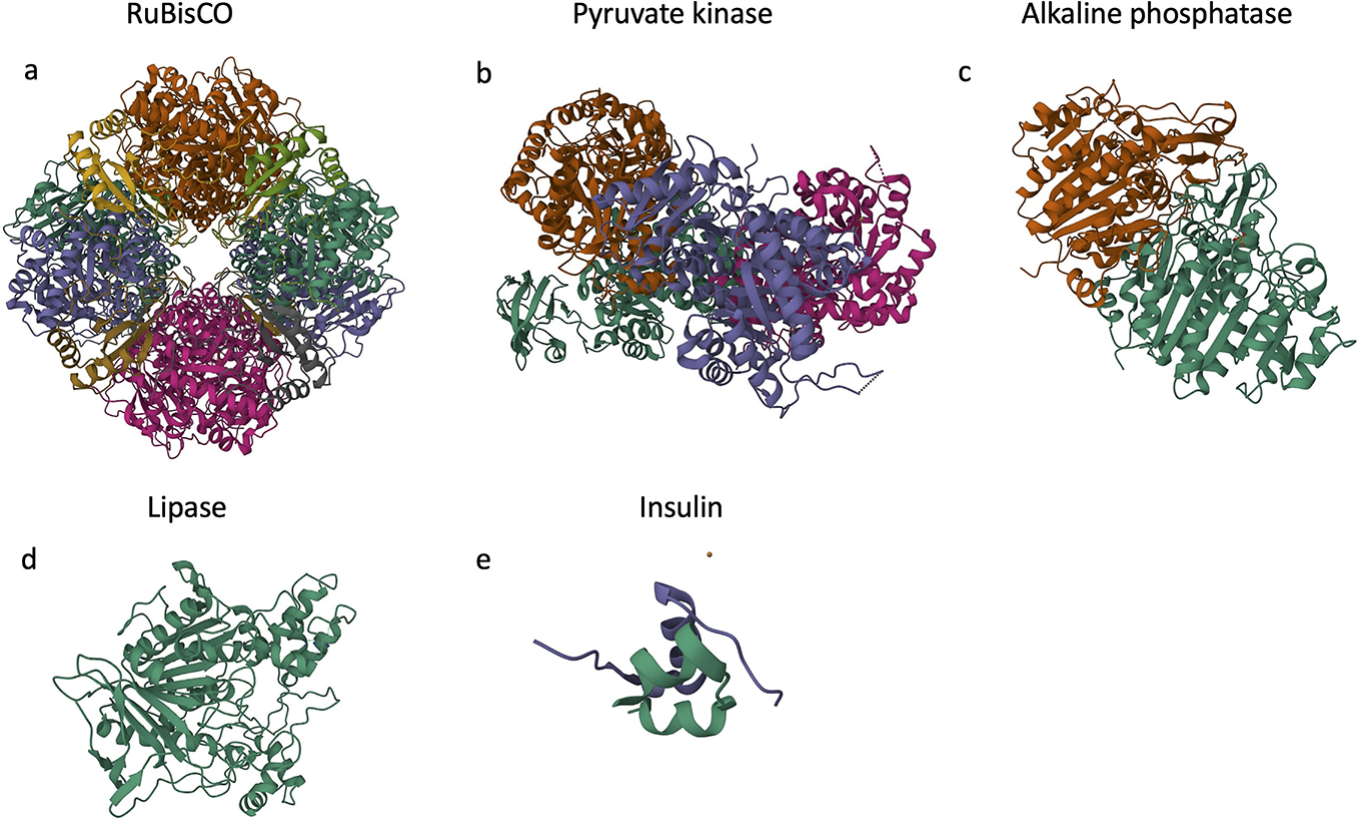
3D structures of proteins investigated in this study from the Research Collaboratory for Structural Bioinformatics (RCSB) Protein Data Bank (PDB) (https://www.rcsb.org/; Berman et al.^42^) of (a) RuBisCO (PDB ID 8RUC; Andersson^43^), (b) pyruvate kinase (PDB ID 7R6Y; Ramirez-Silva et al.^44^), (c) alkaline phosphatase (PDB ID 1ELX; Stec et al.^45^), (d) lipase (PDB ID 1AKN; Wang et al.^46^), and (e) insulin (PDB ID 4M4L; Frankaer et al.^47^). Each protein is shown from a front view. Each color represents a distinct protein subunit. 3D structures were reproduced under the Creative Commons CC0 1.0 Universal Public Domain Dedication.

The molecular weights of the five proteins vary over 3 orders of magnitude, with molecule diameters from 0.435 to 13.2 nm (Table 1). They have a range in structural complexity, with varying degrees of protein folding and number of subunits (Figure 1). For example, RuBisCO and pyruvate kinase have complex quaternary structures with 16 and 4 subunits, respectively, whereas lipase only has a tertiary protein structure with one polypeptide chain. In addition, glutathione was chosen as a simple peptide to compare to proteins. Glutathione is a tripeptide composed of glycine, cysteine, and glutamic acid. It has a molecular weight of 0.303 kDa, 1–3 orders of magnitude smaller than the proteins used in this work. The structures and chemical composition are summarized in Figure 1 and Table 1.

**Table 1:** Protein and Peptide Characteristics

compound	size (kDa)	diameter (nm)	level of protein structure	secondary structural features	reference
RuBisCO	550	13.2	quaternary	β-helix/β-strand/β-turn	Andersson^45^
pyruvate kinase	233	12.5	quaternary	β-helix/β-strand/β-turn	Ramirez-Silva et al.^46^
alkaline phosphatase	140	10.5	quaternary	β-helix/β-strand/β-turn	Stec et al.^47^
lipase	63	0.787	tertiary	β-strand/β-turn	Wang et al.^48^
insulin	5.8	0.435	quaternary	β-helix/β-strand/β-turn	Frankaer et al.^49^
glutathione	0.307	no data	primary	not applicable	not applicable

### Ice Nucleation

Ice nucleation measurements were conducted using a custom-built freezing apparatus optimized from Fornea et al.^48^ to contain a freezing array, enabling the measurement of ice nucleation in 16 samples simultaneously.^49,50^ A 4 × 4 droplet array was made by constructing a spacer using soft poly(dimethylsiloxane) (PDMS, Sylgard 184, Dow Chemical). The droplets (2 μL each) were positioned on a hydrophobic-coated (Rain-X water repellent, ITW Global Brands) glass slide (Fisher Scientific) with another glass slide on top of the PDMS array to seal the droplets in individual compartments in the cooling stage (LTS420, Linkam Scientific Instruments). The cooling stage was cooled from +5 to −40 °C at 0.5 °C min^–1^ for up to 11 freeze–thaw cycles using a temperature controller (T96, Linkam Scientific Instruments) and flow of liquid nitrogen (LNP96, Linkam Scientific Instruments) controlled within LINK software (Linkam Scientific Instruments). Light-emitting diode (LED) lights were attached to the lens of a digital single-lens reflex (DSLR) camera (Canon 5D Mark IV) fitted with a macro lens (Canon EF 100 mm f/2.8) to allow for visualization and imaging. A second camera [charge-coupled device (CCD) camera UI-2240SE-M-GL, IDS Imaging] was used for some measurements as a result of a malfunction of the DSLR camera. An identical resolution (1280 × 1024 pixels) and frame rate were used with both cameras, with images recorded every 6 s, resulting in recorded images every 0.1 °C and a temperature accuracy of the cooling stage of ±0.5 °C.

A three-point temperature calibration was performed to verify accuracy of the recorded temperature for our experiments using the established melting temperatures of *n*-dodecane, *n*-undecane, and *n*-decane.^15,33,48^ Individual calibrations were used for each droplet compartment within the array as a result of temperature gradients across the cooling stage.

The probability of freezing (*P*) or fraction of droplets frozen as a function of the temperature (*T*, °C) was calculated as^51^
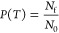
1where *N*_f_ is the cumulative number of droplets frozen at a given temperature (*T*) and *N*_0_ is the total number of freezing events for a sample, compiled from all replicates. The median freezing temperature was reported from the total number of freezing events. The fraction frozen can then be used to calculate the cumulative number of ice nucleation active sites per unit mass of organic sample material at a specific temperature [*n*_m_(*T*)]^8,33,51^
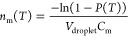
2where *V*_droplet_ is the volume of a single aliquot (2 μL for this study) and *C*_m_ is the mass concentration of an individual peptide or protein in the sample droplet.

### Dynamic Light Scattering (DLS)

DLS is a common method used to detect the size and degree of aggregation of protein solutions.^52,53^ The diffusion coefficient (*D*) is determined by the time-dependent rate of fluctuations in scattered light by particles suspended in solution. The diffusion coefficient is inversely proportional to the hydrodynamic diameter (*D*_h_) of the particles according to the Stokes–Einstein equation, where *k*_B_ is the Boltzmann constant and *R*_h_ is the hydrodynamic radius.^53,54^ For an accurate particle size, the temperature (*T*) and viscosity (η) of the solution must be known.
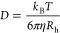
3*D*_h_ is the diameter of a hard sphere with the same diffusion coefficient as the sample, which was determined for each protein and peptide sample at each concentration (from 2 × 10^–1^ to 2 × 10^–5^ mg mL^–1^) using a Zetasizer Nano ZS (Malvern Instruments). Hard spheres are used to describe *D*_h_ because they are used as model particles in light-scattering measurements for calibration. The Zetasizer is fitted with a 632.8 nm laser, and measurements were performed at the standard backscatter angle of 173°, giving *D*_h_ distributions from 0.3 to 1 × 10^4^ nm. The procedural blank used for the ice nucleation measurements was used as a background sample and did not result in any impurities above the limit of detection. Samples were equilibrated inside the instrument at 25 °C for 180 s prior to measurement. Measurements were only recorded when counts of photons in the detector exceeded 20 000 counts per second (20 kcps). Three runs were performed with triplicate samples for a total of nine measurements per sample, giving the scattered light intensity-weighted distributions of protein and peptide samples.

The average particle diameter and number-weighted size distributions were derived from the intensity-weighted distributions directly from the instrument software (version 7.12, Malvern Instruments). The intensity-weighted distributions were converted to number-weighted size distributions to represent the number of molecules in each size bin using the Mie theory with equations for conversion defined elsewhere.^53^ The instrument also provides volume-weighted size distributions, representing the volume of molecules in each size bin. The number-weighted size distributions were converted to the volume-weighted size distributions by assuming the volume of a sphere (4/3π*r*^3^) from the instrument software, where *r* is the radius of a sphere. All data from DLS were normalized directly from the instrument software.

The number of individual protein molecules that can fit into an aggregate was estimated, assuming that the aggregate was a spherical particle with a hydrodynamic diameter of 1 × 10^3^ nm. The number of molecules (*M*) in an aggregate was determined using the following equation:
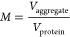
4where *V*_aggregate_ is the volume of the aggregate and *V*_protein_ is the volume of the protein. Protein volumes were estimated by assuming that the protein is a sphere with a hydrodynamic diameter equivalent to the diameters in Table 1.

Monodisperse samples have a polydispersity index (PDI) of <0.1,^53^ but the samples in this study had a broad PDI range of 0.15–1, with most samples being extremely polydisperse (i.e., PDI of >0.7). These calculations assume spherical particles and monodisperse samples. Therefore, the reported average diameter and both number and volume distributions are estimates and should not be used to compare particle sizes to other studies.

## Results and Discussion

**Figure 2:**
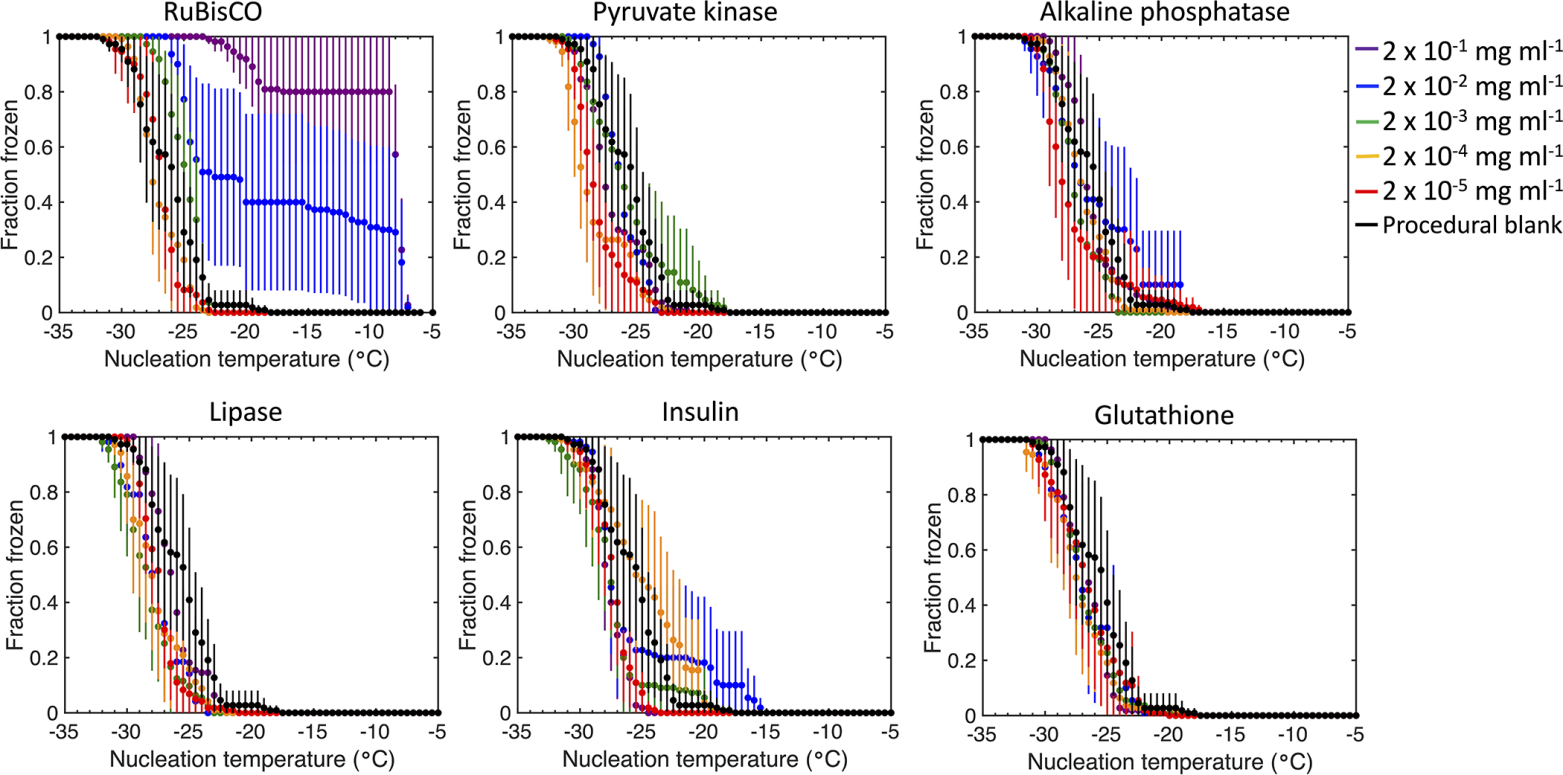
Fraction of droplets frozen as a function of the temperature for each protein and peptide (*n* = 70–110) of (a) RuBisCO, (b) pyruvate kinase, (c) alkaline phosphatase, (d) lipase, (e) insulin, and (f) glutathione at a concentration of 2 × 10^–1^ mg mL^–1^ (purple), 2 × 10^–2^ mg mL^–1^ (blue), 2 × 10^–3^ mg mL^–1^ (green), 2 × 10^–4^ mg mL^–1^ (orange), and 2 × 10^–5^ mg mL^–1^ (red) and the UHPLC water procedural blank (black). Data points represent the mean fraction frozen of the pooled data sets ± the 95% confidence limits.

This study investigated the role of the protein concentration and aggregation as well as molecular size as properties that may contribute to efficient proteinaceous INPs. A total of 10 replicate runs with up to 11 freeze–thaw cycles were performed for each composition and concentration. The fraction frozen (i.e., probability of freezing) was used to visualize the temperature range of freezing events as a result of the stochastic nature of heterogeneous ice nucleation (Figure 2). The error bars shown represent the two-sided 95% confidence level, following the analysis of Worthy et al.^55^ The onset freezing temperature is defined here as the warmest detected freezing temperature, and complete freezing is the temperature at which all droplets are activated as INPs. A summary of the onset, complete, and median nucleation temperatures of each set of replicates is provided in Supporting Table 1 of the Supporting Information.

RuBisCO was an extremely efficient INP with approximately 80% of INPs active at the median freezing temperature of −7.9 °C and a concentration of 2 × 10^–1^ mg mL^–1^ (Figure 2a). In comparison to the other proteins, RuBisCO had the biggest temperature range (19.4 °C) between the onset of freezing (−7.0 °C) and complete freezing (−26.4 °C), which was observed at a concentration of 2 × 10^–2^ mg mL^–1^ (Figure 2a and Supporting Table 1 of the Supporting Information). The shapes of the fraction frozen curves for pyruvate kinase, alkaline phosphatase, and lipase were similar and overlapped at different concentrations (panels b–d of Figure 2). Similarly, the fraction frozen curves for glutathione did not clearly depend upon peptide concentration (Figure 2f).

**Figure 3:**
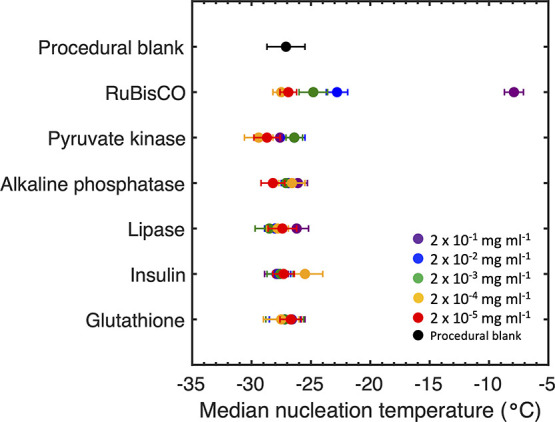
Median freezing temperatures of each protein and peptide at a concentration of 2 × 10^–1^ mg mL^–1^ (purple), 2 × 10^–2^ mg mL^–1^ (blue), 2 × 10^–3^ mg mL^–1^ (green), 2 × 10^–4^ mg mL^–1^ (orange), and 2 × 10^–5^ mg mL^–1^ (red) and the UHPLC water procedural blank (black). Data points show the median ± pooled standard deviation (*n* = 70–110).

Unlike RuBisCO, which is an exceptionally warm nucleator, the remaining samples exhibited fraction frozen behavior closer to that of the procedural blank at many temperatures. Further, to test whether samples possess ice-nucleating ability above that of the blank, additional statistics were performed using JMP Pro 16 statistical software (JMP Statistical Discovery, LLC). The mean freezing temperatures ± pooled standard deviation (*n* = 70–110) are shown in Figure 3. Because the data sets did not meet the requirements of a parametric analysis of variance (ANOVA), a non-parametric one-way ANOVA was performed to test for significant differences between median ice nucleation temperatures using a Kruskal–Wallis test on ranks. Pairwise post hoc comparisons were made with the Wilcoxon test (*p* < 0.05). The results are shown in Supporting Table 2 of the Supporting Information.

RuBisCO had the highest observed median freezing temperature of −7.9 ± 0.8 °C (±pooled standard deviation) at a concentration of 2 × 10^–1^ mg mL^–1^ (Figure 3). The median freezing temperature decreased with a decreasing RuBisCO concentration. In Supporting Table 2 of the Supporting Information, we see that the median freezing temperature of RuBisCO samples was statistically warmer than the median freezing temperature of the procedural blank down to a concentration as low as 2 × 10^–3^ mg mL^–1^. This indicates the presence of some ice-nucleating capacity. For comparison, the median freezing temperature of pyruvate kinase (the other large protein used in this work) was significantly warmer than that of the blank at 2 × 10^–2^ mg mL^–1^. Alkaline phosphatase required a concentration of 2 × 10^–1^ mg mL^–1^ to freeze at a temperature significantly above the blank. Neither lipase nor glutathione samples froze at temperatures above that of the blank at any sampled concentration. Hence, we cannot report any ice-nucleating potential for either of them.

For RuBisCO, a concentration dependence was observed, and each concentration was statistically different from the next at the 95% confidence level, except for the lowest two concentrations (2 × 10^–4^ and 2 × 10^–5^ mg mL^–1^; Supporting Table 2 of the Supporting Information). Similar concentration trends were not observed for the other samples. In fact, for a reason that is not clear, insulin at a low concentration (2 × 10^–4^ mg/mL) froze at temperatures significantly above the blank, but no other concentration did.

Glutathione was used as a non-aggregating control for this study because it is a simple tripeptide (composed of glutamic acid, cysteine, and glycine) with only a primary structure composed of a linear sequence of the three amino acids.^56^ Glutathione had a narrow range of median freezing temperatures from −26.6 to −27.5 °C with no statistical difference between concentrations (Figure 3). The freezing temperature of glutathione at 2 × 10^–1^ mg mL^–1^ (−26.6 ± 1.1 °C) was statistically the same as that of pyruvate kinase, alkaline phosphatase, or lipase at the same concentration.

The molecular size of proteins has previously been used as an indicator of ice nucleation activity.^40,57−59^ The size of a protein has been shown to control the kinetics of water crystallization, with small proteins (<10 kDa) acting as antifreeze proteins and large proteins (>100 kDa) acting as efficient INPs.^3,35,58^ In this study, the largest protein (RuBisCO at 550 kDa) was the most ice-active, and the smallest protein (insulin at 5.8 kDa) was the weakest INP, which seemingly supports this hypothesis. However, only RuBisCO was an efficient INP, and the relationship between the protein size and INP efficiency was nonlinear from the proteins investigated in this study. Therefore, the molecular size may not be suitable for parametrization of proteinaceous biological INPs, as previously suggested.^35^ Our results demonstrate that more research is needed to include freezing temperature data across a broad size range of proteinaceous material before parametrizations should be considered.

**Figure 4:**
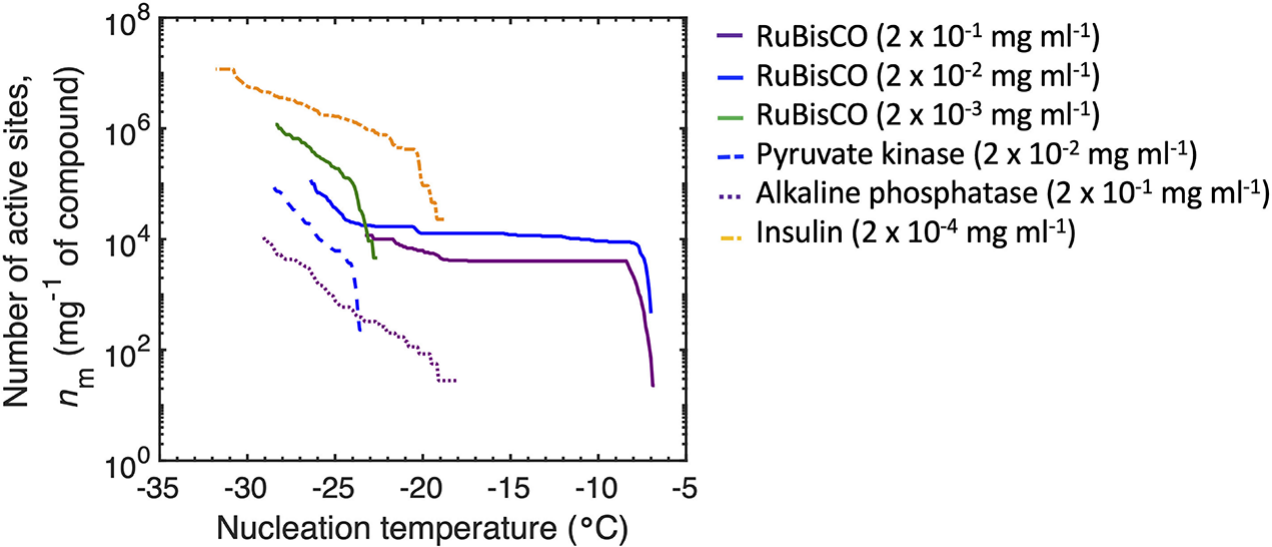
Cumulative number of active sites per mass in milligrams as a function of the temperature [*n*_m_(*T*)] (*n* = 70–110) of RuBisCO (solid line) at a concentration of 2 × 10^–1^ mg mL^–1^ (purple), 2 × 10^–2^ mg mL^–1^ (blue), and 2 × 10^–3^ mg mL^–1^ (green), pyruvate kinase (dashed line) at a concentration of 2 × 10^–2^ mg mL^–1^ (blue), alkaline phosphatase (dotted line) at a concentration of 2 × 10^–1^ mg mL^–1^ (purple), and insulin (dotted–dashed line) at a concentration of 2 × 10^–4^ mg mL^–1^ (orange). Only samples that were statistically warmer than the procedural blank are shown.

On a given INP, the location where ice nucleation preferentially occurs is termed an active site. The ice nucleation active site density or number of active sites^60^ is calculated from the fraction frozen using the droplet volume of a single aliquot (2 μL for this study) and the mass concentration of an individual protein in the sample droplet, according to eq 2. The cumulative number of active sites per mass of protein is presented in Figure 4. Only samples in which the median nucleation temperature was warmer than the blank by a statistically significant amount are included. RuBisCO had the highest number of ice nucleation active sites per mass (*n*_m_) at the warmest nucleation temperature of any protein in this study. RuBisCO had the highest density of active sites at −7 °C with 1.8 × 10^3^ mg^–1^ and over 3 times as many active sites at −20 °C with a *n*_m_ of 6.3 × 10^3^ mg^–1^ at a concentration of 2 × 10^–1^ mg mL^–1^ (Figure 4). However, at temperatures of < –20 °C, insulin had the greatest site density with 1.2 × 10^7^ active sites compared to 1.2 × 10^4^ active sites for RuBisCO. All five proteins had a similar number of ice nucleation active sites as RuBisCO at a concentration of 2 × 10^–1^ mg^–1^ but only at relatively cold temperatures (< –25 °C) (Figure 4).

For RuBisCO, the number of ice nucleation active sites increased with a decreasing protein concentration but was associated with lower nucleation temperatures and less efficient INPs. Similarly, a low concentration of less than 10^–5^ μg μL^–1^ InaZ from *P. syringae* led to freezing temperatures of < –20 °C,^38^ which is in the range of low freezing temperatures observed here for RuBisCO concentrations less than 2 × 10^–2^ mg mL^–1^. The lower nucleation temperatures of InaZ are thought to be attributed to the absence of protein aggregates and a subsequent lower number of ice nucleation sites at a low concentration.^38^ Further work is needed to determine if the ice nucleation active sites present at lower freezing temperatures for proteins (pyruvate kinase, alkaline phosphatase, and insulin) in this study are potentially composed of structures with relatively inefficient chemical bonding for water crystallization.

**Figure 5:**
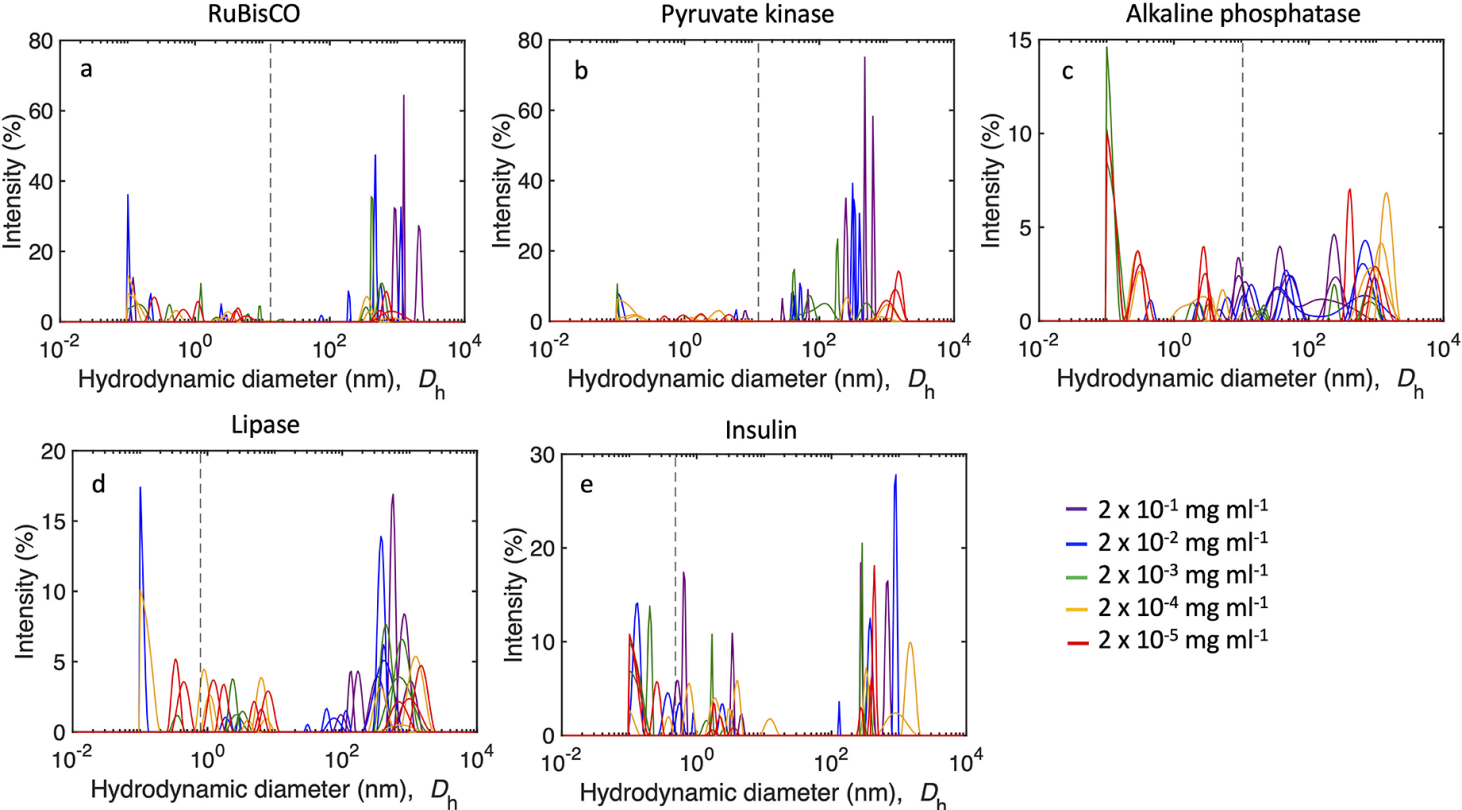
DLS spectra of protein solutions. Hydrodynamic diameter (*D*_h_) distribution by intensity of (a) RuBisCO, (b) pyruvate kinase, (c) alkaline phosphatase, (d) lipase, and (e) insulin at a concentration of 2 × 10^–1^ mg mL^–1^ (purple), 2 × 10^–2^ mg mL^–1^ (blue), 2 × 10^–3^ mg mL^–1^ (green), 2 × 10^–4^ mg mL^–1^ (orange), and 2 × 10^–5^ mg mL^–1^ (red). The dashed black line refers to the diameter of the protein molecule. Glutathione is too small to be detected by DLS. Note that the *y*-axis scale is different for each graph.

Protein size distributions were determined by DLS, which is a technique used to determine the size distribution based on the Brownian motion of particles in solution.^53^ This technique allowed us to investigate a range of proteins with varying the molecular size and concentration over 5 orders of magnitude. DLS was chosen as a result of the ability to detect large aggregates in samples over a broad range in diameter with multiple populations of particles.^52,53^ The proteins had a wide range of detected hydrodynamic diameters (*D*_h_), from approximately 0.1 to over 1000 nm (Figure 5). Glutathione is absent from Figure 5 because it is not detectable using DLS; the individual molecules are smaller than the limit of detection of DLS (<0.3 nm), and glutathione did not aggregate into larger structures. The presence of both fragmentation and aggregation of RuBisCO with diameters from <0.1 to >10^3^ nm across a range of concentrations was observed in the intensity-weighted size distribution (i.e., scattering light intensity of particles at a given diameter) (Figure 5a). The largest measured *D*_h_ of RuBisCO (2.9 × 10^3^ ± 3.4 × 10^2^ nm; mean ± standard deviation) was associated with the highest median nucleation temperature of −7.9 °C, at a concentration of 2 × 10^–1^ mg mL^–1^. In addition, the number-weighted size distribution demonstrated that >60% of aggregated RuBisCO molecules at 2 × 10^–1^ mg mL^–1^ had *D*_h_ of >10^3^ nm (Supporting Figure 2 of the Supporting Information). At a lower RuBisCO concentration, the median freezing temperature was lower (ranging from −22.8 to −27.5 °C) and the concentration of large aggregates was reduced, with an absence of large aggregates below 2 × 10^–1^ mg mL^–1^ (Figure 5a). An increase in fragmentation of RuBisCO occurred at concentrations of <2 × 10^–1^ mg mL^–1^, with >30% of particles detected with *D*_h_ less than the size of one RuBisCO molecule. Large aggregating particles were detected for the remaining four proteins in the intensity-weighted size distributions with *D*_h_ of up to 2 × 10^3^ nm for lipase, similar to RuBisCO (Figure 5).

**Figure 6:**
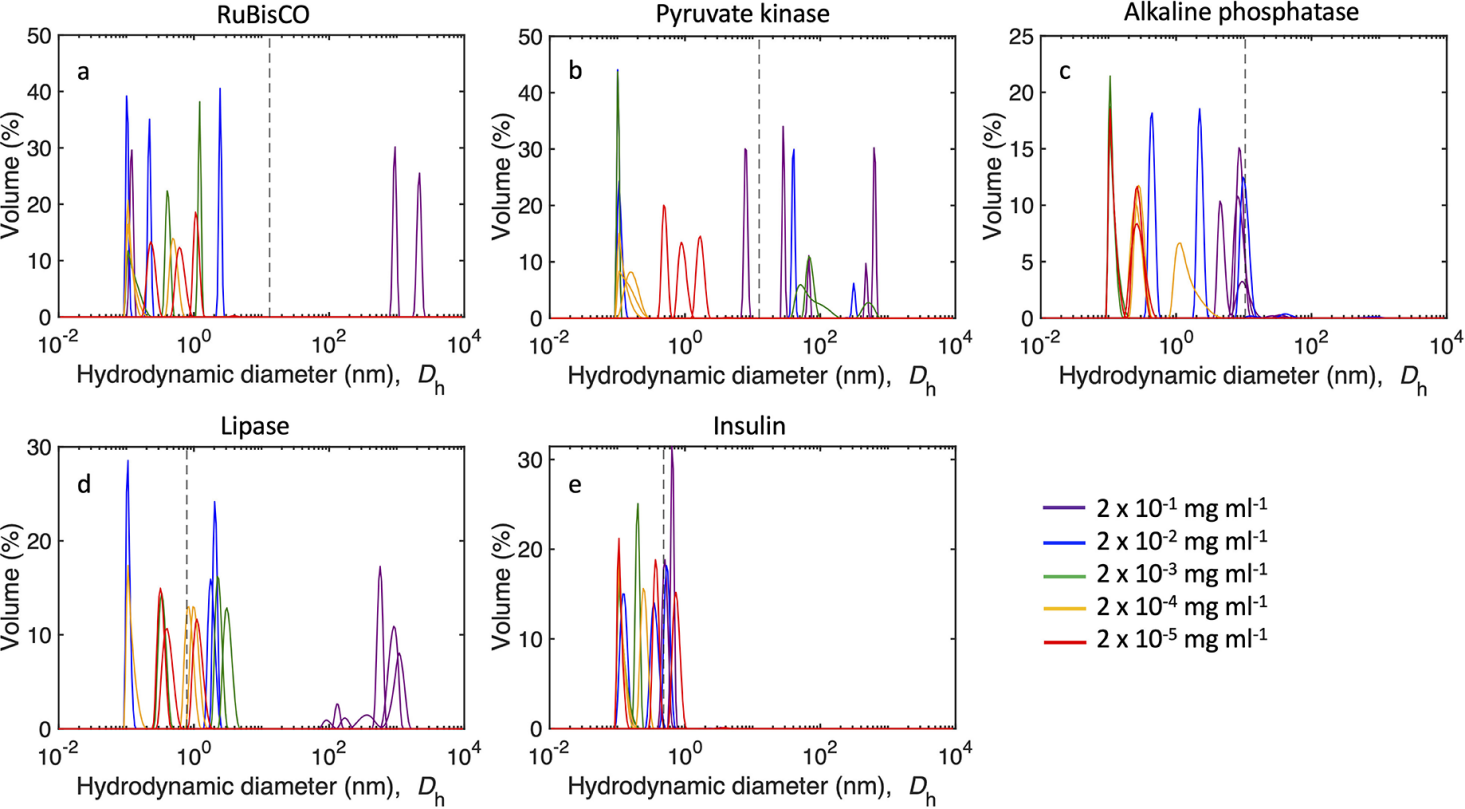
DLS spectra of protein solutions. Hydrodynamic diameter (*D*_h_) distribution by volume-derived intensity of (a) RuBisCO, (b) pyruvate kinase, (c) alkaline phosphatase, (d) lipase, and (e) insulin at a concentration of 2 × 10^–1^ mg mL^–1^ (purple), 2 × 10^–2^ mg mL^–1^ (blue), 2 × 10^–3^ mg mL^–1^ (green), 2 × 10^–4^ mg mL^–1^ (orange), and 2 × 10^–5^ mg mL^–1^ (red). The dashed black line refers to the diameter of the protein molecule. Glutathione is too small to be detected by DLS. Note that the *y*-axis scale is different for each graph.

The volume-weighted size distribution (i.e., the total volume of particles at a given diameter) from DLS measurements gives a more quantitative assessment of the particle size distribution compared to the intensity-weighted distribution. The volume-weighted size distribution showed that >60% of the total RuBisCO volume occurred at a diameter from approximately 1 to 2 × 10^3^ nm at the highest protein concentration of 2 × 10^–1^ mg mL^–1^ (Figure 6a). At lower RuBisCO concentrations, the volume-derived intensity indicated that particles had *D*_h_ of <10^1^, which is smaller than the size of individual RuBisCO molecules (diameter of a RuBisCO molecule is 13.2 nm; Table 1). Approximately 30% of the volume at the highest concentration of RuBisCO (2 × 10^–1^ mg mL^–1^) contained particles with *D*_h_ of 10^–1^ nm (Figure 6a), indicative of fragmented RuBisCO.

The total number of molecules in a protein aggregate was calculated by assuming that the aggregate was a spherical particle with a hydrodynamic diameter of 10^3^ nm (eq 4). The number of aggregated molecules (*M*) in a 10^3^ nm diameter aggregate of RuBisCO was 4.3 × 10^5^ molecules. Aggregates for large protein diameters were insignificant in the volume-weighted size distributions for all other proteins, except for lipase, which contained peaks of >10^3^ nm with a volume fraction over 60% at the highest protein concentration (2 × 10^–1^ mg mL^–1^), similar to RuBisCO (Figure 6). The number of lipase molecules in an aggregate with a diameter of 10^3^ nm was approximately 2.1 × 10^9^ molecules (eq 4). Therefore, the number of molecules making up a 10^3^ nm aggregate is very different for each protein as a result of a large difference in the diameter of a single protein molecule, with *M*_lipase_ containing almost 4 orders of magnitude additional protein molecules than *M*_RuBisCO_. For the remaining proteins, peak volume-weighted size distributions were observed at much smaller diameters (Figure 6). Pyruvate kinase had peak diameters from ∼10^–1^ to 10^2^ nm, indicating both fragmentation and aggregation occurring at a range of concentrations (Figure 6). Alkaline phosphatase had peak volume-weighted size distributions of <10^1^ nm, indicating mostly fragmentation of this protein (Figure 6c). Insulin had peak volume-weighted size distributions of <10^–1^ nm (Figure 6e). However, insulin is also the smallest protein measured in this study (0.435 nm), and therefore, aggregates were still present at all concentrations.

Both the particle size and concentration of INPs in atmospheric droplets have been suggested to influence the freezing temperature.^3^ The change in ice nucleation efficiency for biological material over a range in concentration has largely been overlooked until recently.^26,30,61^ Decreasing the concentration of two large proteins, apoferritin (440–500 kDa) and ferritin (481 kDa), reduced ice nucleation efficiency, which was attributed to disaggregation or disassembly of proteins into individual subunits.^26^ The variability in the observed freezing temperature in our study may be partially explained by the wide range of aggregation and disassembly behaviors of the proteins, as indicated by the DLS spectra. Our DLS measurements show that the protein molecular size is not a good predictor of protein aggregation, with a range of aggregate and fragment sizes present in solutions of proteins ranging in molecular mass from 5.8 to 550 kDa. This variability was affected by the protein concentration, although not predictably. While the highest protein concentrations resulted in larger aggregates in both RuBisCO and lipase, it was only for RuBisCO that there was a significant increase in the median freezing temperature at a concentration of 2 × 10^–1^ mg mL^–1^ compared to the concentration of 2 × 10^–2^ mg mL^–1^ and lower. Our data are consistent with previous work^33^ at a higher concentration of RuBisCO (5 × 10^–1^ mg mL^–1^), which had an even warmer freezing temperature than observed in the current work (Supporting Figure 3 of the Supporting Information). This suggests that the high concentration of RuBisCO by Alsante et al.^33^ was aggregated, although this was not measured. We showed both lipase and pyruvate kinase had significantly lower freezing temperatures than the procedural blank with a decreasing protein concentration, but the large variability in the freezing temperature and warm freezing onset suggests that they should not be considered antifreeze proteins.

Protein aggregates exhibit a wide range of morphology, including both amyloid fibrils (ordered planar aggregates) and particulates (irregular and spherical).^62^ Although the aggregation behavior of RuBisCO is largely unexplored, a recent study determined that RuBisCO may aggregate into fibrils with a lattice-like structure within carboxysomes (specialized microcompartments for carbon fixation within autotrophic bacterial cells).^63^ All other proteins investigated in this study (i.e., pyruvate kinase, alkaline phosphatase, lipase, and insulin) are known to aggregate into amyloid fibrils.^64−67^ Other proteins known to be efficient INPs at temperatures of > –10 °C, such as InaZ from *P. syringae* and ferritin, are known to aggregate as well.^26,38^ InaZ from *P. syringae* is suspected to aggregate into planar-like structures from aggregated β-helical structures.^68,69^ Modeling indicates that the ice-active site within InaZ of *P. syringae* is a β-helical structure with an amino acid sequence composed of a TxT motif, where T is threonine and x is a non-conserved amino acid.^68,70^ These TxT motifs order water molecules into a crystalline lattice structure attributed to chemical functional groups, including hydroxyl and amine groups, capable of hydrogen bonding to water molecules composed of hydrophilic–hydrophobic patterns.^35,71,72^ Therefore, increasing aggregated β-helical structures may increase the ordering of water molecules into a crystalline lattice structure.^3,35,71,72^ However, the number and alignment of INPs within the aggregate as well as the three-dimensional (3D) structure of the protein is still unknown.^38^

The high ice nucleation activity of RuBisCO may be attributed to a distinct aggregate morphology. Previously, heat denaturation showed that the 3D structure (secondary, tertiary, or quaternary) of RuBisCO was essential for its high ice nucleation activity at temperatures warmer than −10 °C but not necessary at freezing temperatures less than −20 °C.^33^ Although other weakly efficient proteinaceous INPs contain quaternary structures (i.e., pyruvate kinase, alkaline phosphatase, and insulin), RuBisCO is the most structurally complex protein in this study, with eight copies of a large subunit (51–58 kDa) and eight copies of a small subunit (12–18 kDa), potentially increasing the ice active surface area of this protein.^33^ Future work should characterize the physicochemical features of RuBisCO and other INPs, resulting in highly efficient nucleation temperatures.

Proteins are known to be enriched in the atmosphere and may come from sea spray, soils, and terrestrial plants.^73−76^ Whole pollen grains contain 2.5–61% protein by mass.^77^ Aerosolized subpollen particles (SPPs) released from pollen grains have high atmospheric concentrations^78^ and are efficient biogenic sources of INPs with a high protein concentration (from 2.4 × 10^–3^ to 0.7 × 10^–2^ mg mL^–1^).^10^ Ice nucleation activity decreases with a decreasing protein content.^11^ Soil may contain up to 30 mg of protein per gram,^79^ which includes a significant contribution from RuBisCO-containing soil microalgae.^80^ Proteins are enriched up to 120 000 times in sea spray aerosol (SSA) compared to bulk seawater.^81^ Although lipase is the only individual protein known to be enriched in SSA,^82^ observed freezing temperatures in this study indicate it likely does not contribute to efficient INPs. RuBisCO is present in the surface seawater at concentrations of 2 × 10^–5^ mg mL^–1^ and, therefore, may be enriched in the atmosphere at concentrations as high as 2.4 mg mL^–1^, assuming that it was enriched in SSA 120 000 times compared to the surface seawater concentration.^79,83^ These potentially high concentrations of atmospheric RuBisCO in SSA may result in aggregation and lead to highly efficient INPs. Because it is difficult to envision how RuBisCO from terrestrial plant leaves enters the atmosphere, SSA may represent a significant source of aerosolized RuBisCO.

A decreasing ice nucleation activity was associated with a decreasing number of molecules in RuBisCO aggregates as well as fragmentation into larger numbers of individual subunits or other smaller particles. The four additional proteins aggregated in solution but were only weakly effective INPs, regardless of the concentration spanning 5 orders of magnitude (from 2 × 10^–1^ to 2 × 10^–5^ mg mL^–1^) and diameter ranging from approximately 0.1 to over 10^3^ nm. It is also possible that aggregation affects the ice nucleation of different proteins in different ways. In the case of RuBisCO, aggregation at high concentrations resulted in an increase in ice nucleation efficiency. However, aggregation was observed for all proteins in this study, but aggregation did not always increase the ice nucleation efficiency. Fragmentation was observed for all of the proteins investigated in this study.

Fragmentation is caused by a disruption in a covalent bond and may occur spontaneously through hydrolysis of susceptible amino acids, such as aspartic acid and tryptophan.^84,85^ Fragmentation susceptibility in solution occurs as a result of the flexibility of the tertiary structure backbone and side chains of amino acids, resulting in peptide bond cleavage.^85^ Solvent conditions (extreme pH and temperature) can facilitate cleavage of the protein, which may result in lower ice nucleation efficiency observed for apoferritin at acidic conditions.^26,84^ Studies have shown that acidity is likely to change the structure of proteins.^84,86^ Given this fact, changes in aggregation and ice nucleation as a function of pH would be a valuable subject for future work.

Although amino acid composition affects the aggregation of proteins, individual amino acids and peptides do not aggregate on their own.^87^ The freezing temperature of the peptide (glutathione) was not statistically different at a range of concentrations (from 5 × 10^–2^ to 5 × 10^–5^ mg mL^–1^) with a narrow range in the median freezing temperature (from −26.6 to −27.5 °C). In addition, the freezing temperature of glutathione was similar to the immersion mode nucleation temperature (−21.8 ± 2.2 °C; mean ± SD) observed in a previous study at a higher concentration (5 × 10^–1^ mg mL^–1^).^33^ The narrow range in freezing temperature across a broad range of concentrations could potentially be a result of an absence in aggregation of the peptide, which was confirmed by the absence of peaks when the solution was measured using DLS. A range of chemically diverse individual amino acids were shown to be INPs in a previous study, with no relationship between chemical functional groups and INP efficiency.^33^ The mean freezing temperature of glycine and cysteine, both components of glutathione, were −20.5 ± 2.3 and −20.5 ± 2.92 °C, respectively.^33^ The ice nucleation activity of glutathione and individual amino acids indicates that the aggregation of biomolecules is not essential for ice nucleation to occur.

As a reflection of the molecular mass, there were significant differences in the lengths of the amino acid sequences among the different proteins. The smallest protein in this study, bovine insulin, consists of 51 amino acids arranged in two chains (Figure 1),^88^ whereas the 16 subunits of spinach RuBisCO form a protein of ∼5240 amino acids^33^ (Figure 1). The amino acid sequence of RuBisCO did not show any aggregating hydrophilic–hydrophobic motifs that are similar to other currently known INPs.^33^ With the exception of insulin, the relative frequencies of individual amino acids as well as groups of amino acids (on the basis of the structure and function) were similar in RuBisCO and the other proteins used in this study, suggesting that the relative abundance of the amino acids in the primary structure was not a factor determining the high freezing temperatures observed for RuBisCO (Supporting Figures 4 and 5 of the Supporting Information). Some amino acids were notably absent from insulin (Supporting Figure 4 of the Supporting Information), which can be related to the small size of this protein. Cysteine has a higher frequency of occurrence in insulin compared to the other proteins (Supporting Figure 4 of the Supporting Information) and is known to be a moderately efficient INP.^33^ However, insulin did not have a high ice nucleation efficiency; therefore, we cannot relate INP efficiency to relative individual amino acid frequency within proteins.

Our results, in addition to several recent papers,^26,33−35,58,69^ demonstrate that a wide range of proteins, in terms of the size, structure, and biological function, promote ice nucleation. This study confirms previous work that the abundant and ubiquitous enzyme RuBisCO is an effective ice nucleus at relatively warm temperatures.^33^ The freezing of droplets containing RuBisCO, in terms of both onset and complete freezing, was dependent upon the concentration of RuBisCO. Higher concentrations of RuBisCO affected ice nucleation at warmer temperatures. Enhanced aggregation into large aggregates was observed at relatively high concentrations of RuBisCO, indicating that protein aggregation affected ice nucleation. Large molecular sizes and aggregation were not indicators of efficient ice nucleation for the other proteins used in this study. Consequently, large molecular size and aggregation are not universal indicators of efficient protein INPs. Further work is needed to understand how the morphology and aggregation behavior of proteins affect ice nucleation, with the goal of understanding molecular level mechanisms of ice nucleation catalyzed by proteinaceous INPs.

## Supporting Information Available

Immersion mode ice nucleation data (Supporting Table 1), non-parametric ANOVA of the nucleation temperature (Supporting Table 2), freezing temperature of RuBisCO (2 × 10^–1^) and the procedural blank over repeated freeze–thaw cycles (Supporting Figure 1), hydrodynamic diameter distribution by number-derived intensity (Supporting Figure 2), fraction of droplets frozen of RuBisCO from this study from 2 × 10^–1^ to 2 × 10^–5^ mg mL^–1^ and Alsante et al.^33^ at 5 × 10^–1^ mg mL^–1^ (Supporting Figure 3), relative frequency of the 20 amino acids from each protein (Supporting Figure 4), and relative frequency of amino acid functional groups for each protein (Supporting Figure 5) (PDF)

## Abbreviations

INP: ice-nucleating particle
RuBisCO: ribulose-1,5-bisphosphate carboxylase/oxygenase
SSA: sea spray aerosol
DLS: dynamic light scattering
SPP: subpollen particle.3
