# Supplementary material for: Effect of Aggregation and Molecular Size on the Ice Nucleation Efficiency of Proteins
Source: Environ Sci Technol. 2024 Feb 26;58(10):4594–605. doi: 10.1021/acs.est.3c06835 (PMC10938890; doi:10.1021/acs.est.3c06835)
Supplement: Supplementary file 1 — es3c06835_si_001.pdf [file es3c06835_si_001.pdf]

Effect of aggregation on the ice nucleation efficiency of RuBisCO and other proteins

Alyssa N. Alsante[1†], Daniel C.O. Thornton[1], Sarah D. Brooks[2*]*

[1]Department of Oceanography, Texas A&M University, College Station, TX, United States

[2]Department of Atmospheric Sciences, Texas A&M University, College Station, TX, United States

[†]Present address: Environmental Sciences Division, Oak Ridge National Laboratory, Oak Ridge, TN, 37830, United States

[*]Corresponding author: Daniel C.O. Thornton (dthornton@tamu.edu)

Supporting Information (SI)

Number of pages: 8
Number of tables: 2
Number of figures: 5

Supporting Table 1. Immersion mode ice nucleation. Median (± pooled standard deviation) immersion mode nucleation temperature of each protein and peptide. Onset freezing indicates the warmest detected freezing temperature and complete freezing is the temperature at which all droplets are frozen. Replicates is the number of independent samples analyzed. Total data points indicates the total number of freezing temperatures obtained by pooling the data for all independent replicates.

**Compound**	**Concentration (mg ml^{-1})**	**Replicates**	**Total Data points**	**Onset freezing** (°C)	**Complete freezing** (°C)	**Median nucleation temperature (T$_{50}$, °C)**
Procedural blank (uHPLC water)	No data	10	110	-18.3	-31.9	-27.1 ± 1.6
RuBisCO	2 x 10^{-1}	10	110	-6.8	-23.3	-7.9 ± 0.8
RuBisCO	2 x 10^{-2}	10	110	-7.0	-26.4	-22.8 ± 0.9
RuBisCO	2 x 10^{-3}	10	110	-22.6	-28.4	-24.8 ± 1.2
RuBisCO	2 x 10^{-4}	10	110	-23.3	-30.1	-27.5 ± 0.7
RuBisCO	2 x 10^{-5}	10	110	-23.0	-31.6	-26.9 ± 0.7
Pyruvate kinase	2 x 10^{-1}	10	110	-20.8	-30.7	-27.6 ± 1.2
Pyruvate kinase	2 x 10^{-2}	10	110	-23.4	-28.7	-26.4 ± 0.9
Pyruvate kinase	2 x 10^{-3}	10	110	-17.7	-30.6	-26.4 ± 0.7
Pyruvate kinase	2 x 10^{-4}	10	110	-22.1	-32.0	-29.4 ± 1.2
Pyruvate kinase	2 x 10^{-5}	10	110	-23.2	-31.5	-28.7 ± 1.1
Alkaline phosphatase	2 x 10^{-1}	10	90	-18.1	-29.2	-26.1 ± 0.8
Alkaline phosphatase	2 x 10^{-2}	10	90	-18.0	-31.4	-26.9 ± 0.6
Alkaline phosphatase	2 x 10^{-3}	10	90	-23.5	-30.7	-27.1 ± 0.8
Alkaline phosphatase	2 x 10^{-4}	10	110	-19.9	-30.8	-26.6 ± 1.1
Alkaline phosphatase	2 x 10^{-5}	10	110	-16.9	-30.6	-28.2 ± 1.0
Lipase	2 x 10^{-1}	10	70	-21.3	-29.5	-26.2 ± 1.0
Lipase	2 x 10^{-2}	10	70	-24.0	-32.2	-28.0 ± 0.9
Lipase	2 x 10^{-3}	10	70	-23.1	-32.2	-28.5 ± 1.2
Lipase	2 x 10^{-4}	10	90	-22.2	-31.1	-27.8 ± 0.9
Lipase	2 x 10^{-5}	10	90	-21.1	-30.2	-27.4 ± 1.2
Insulin	2 x 10^{-1}	10	110	-24.8	-31.1	-27.9 ± 1.0
Insulin	2 x 10^{-2}	10	110	-15.4	-31.3	-27.7 ± 1.0
Insulin	2 x 10^{-3}	10	110	-18.1	-32.6	-27.6 ± 1.1
Insulin	2 x 10^{-4}	10	110	-18.8	-31.8	-25.5 ± 1.5
Insulin	2 x 10^{-5}	10	110	-23.9	-31.2	-27.3 ± 0.9
Glutathione	2 x 10^{-1}	10	110	-19.2	-29.7	-26.6 ± 1.1
Glutathione	2 x 10^{-2}	10	110	-20.2	-31.2	-27.2 ± 1.3
Glutathione	2 x 10^{-3}	10	110	-19.8	-31.1	-27.2 ± 1.6
Glutathione	2 x 10^{-4}	10	110	-18.8	-31.9	-27.5 ± 1.5
Glutathione	2 x 10^{-5}	10	110	-20.0	-31.3	-26.7 ± 0.9

Supporting Table 2. Non-parametric ANOVA of nucleation temperature. Non-parametric ANOVA performed using Kruskal-Wallis test on ranks (H = 865.89, degrees of freedom = 30, $p < 0.0001$). Post-hoc analysis (Wilcoxon method) was used to determine whether there was a significant difference in the median ice nucleation temperature between solutions of organic compounds, or between solutions of organic compounds and the procedural blank (uHPLC water). Green boxes indicate the two sample types were significantly different ($p < 0.05$) and red boxes indicate the two sample types were not significantly different ($p > 0.05$). Blue boxes indicate that the freezing temperature of the protein sample was statistically colder ($p < 0.05$) than the procedural blank (uHPLC).

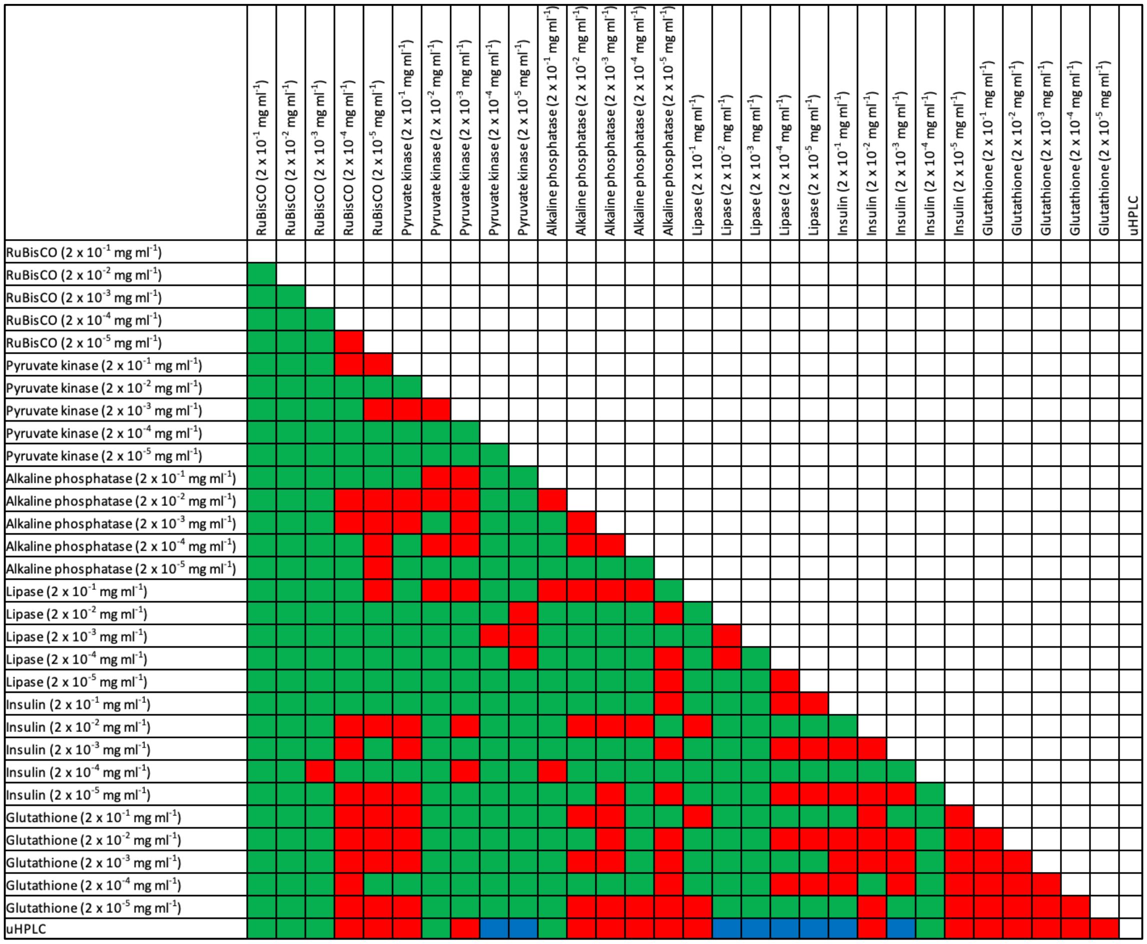

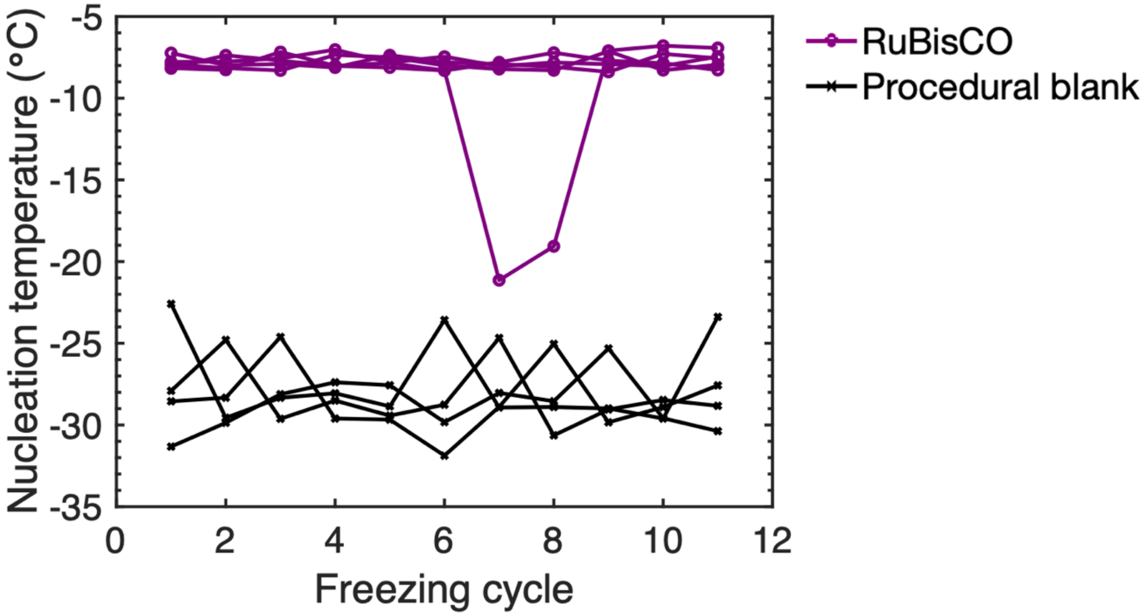

Supporting Figure 1. Observed freezing temperatures over repeated 11 freeze-thaw cycles from five replicates of RuBisCO (purple) at 2 x 10^{-1} mg ml^{-1} and the uHPLC water procedural blank (black).

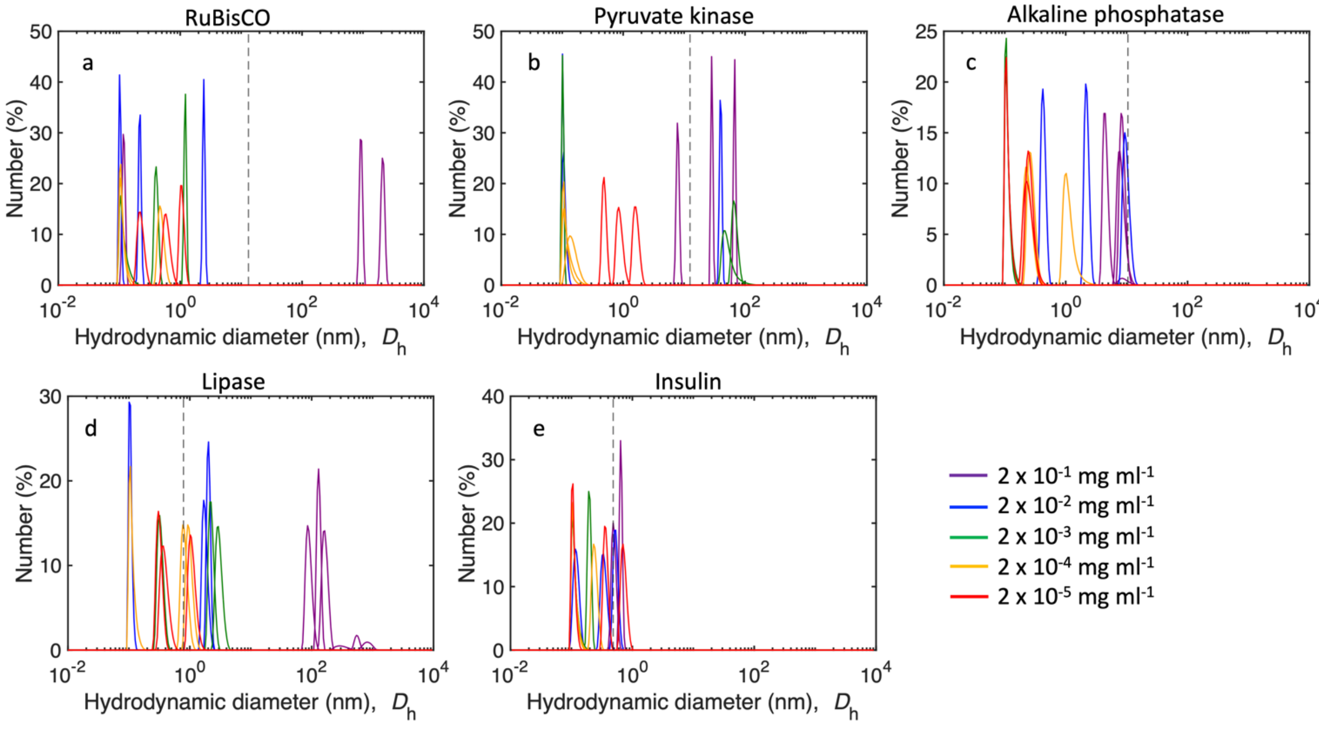

Supporting Figure 2. Hydrodynamic diameter (D_h) distribution by number derived intensity of (**a**) ribulose-1,5-carboxylase/oxygenase (RuBisCO), (**b**) pyruvate kinase, (**c**) alkaline phosphatase, (**d**) lipase, and (**e**) insulin at a concentration of 2 x 10^{-1} mg ml^{-1} (purple), 2 x 10^{-2} mg ml^{-1} (blue), 2 x 10^{-3} mg ml^{-1} (green), 2 x 10^{-4} mg ml^{-1} (orange), and 2 x 10^{-5} mg ml^{-1} (red). The dashed black line refers to the diameter of the protein molecule.

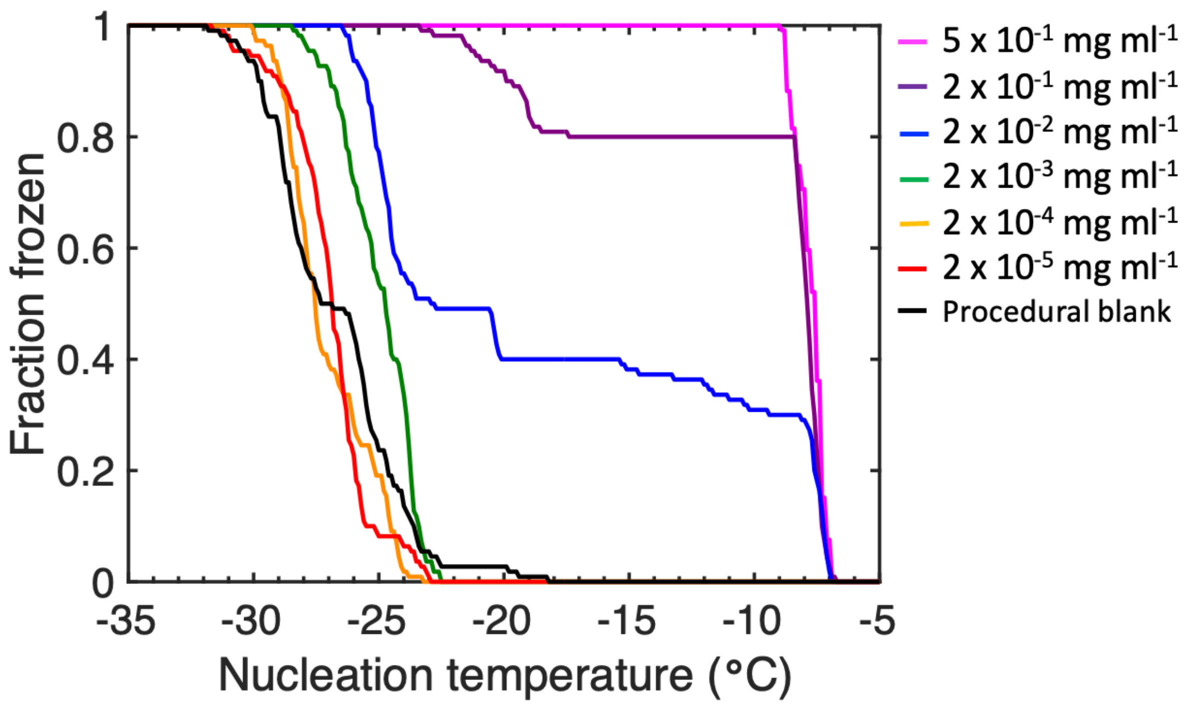

Supporting Figure 3. Fraction of droplets frozen as a function of temperature for ribulose-1,5-carboxylase/oxygenase (RuBisCO) (n = 70-119) at a concentration of 5 x 10^{-1} mg ml^{-1} (pink), 2 x 10^{-1} mg ml^{-1} (purple), 2 x 10^{-2} mg ml^{-1} (blue), 2 x 10^{-3} mg ml^{-1} (green), 2 x 10^{-4} mg ml^{-1} (orange), 2 x 10^{-5} mg ml^{-1} (red), and the uHPLC water procedural blank (black). The RuBisCO data at 5 x 10^{-1} mg ml^{-1} was used from Alsante et al. (2023) according to Creative Commons Attribution 4.0 International License.

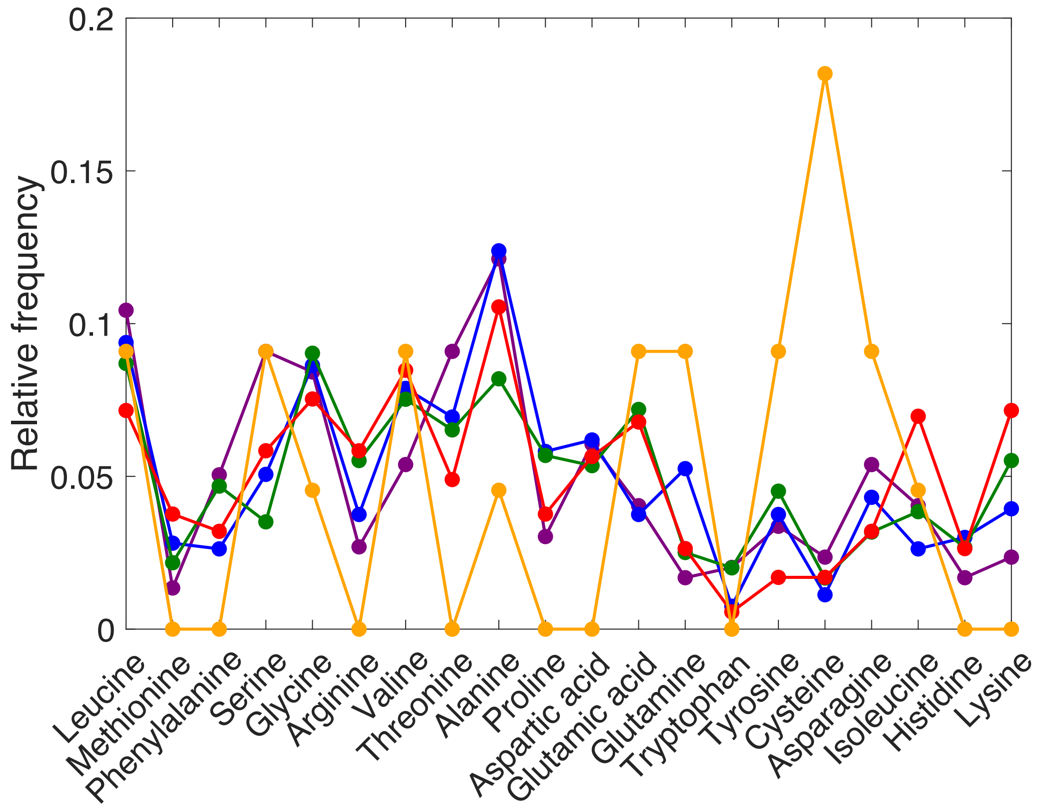

Supporting Figure 4. Relative frequency of the 20 amino acids from ribulose-1,5-carboxylase/oxygenase (RuBisCO) (green), pyruvate kinase (red), alkaline phosphatase (blue), lipase (purple), and insulin (orange).

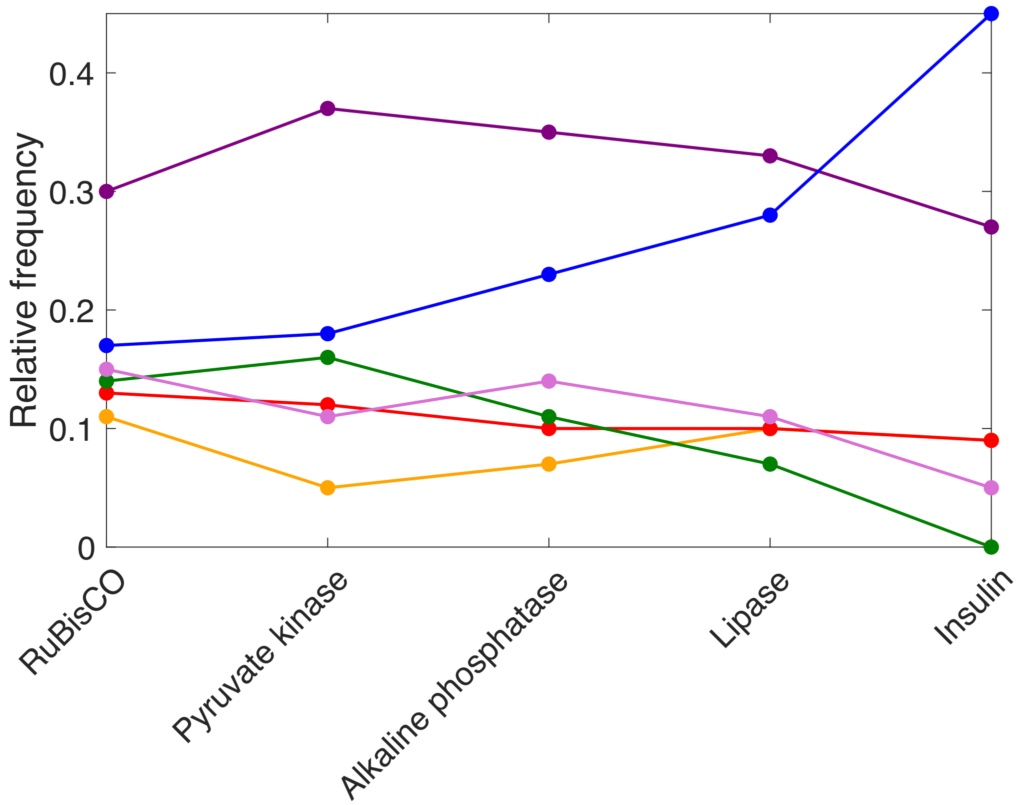

Supporting Figure 5. Relative frequency of aliphatic (purple), aromatic (orange), neutral (blue), acidic (red), basic (green), and other (light purple) amino acids from each protein.
